# Hypertrophy-Promoting Effects of Leucine Supplementation and Moderate Intensity Aerobic Exercise in Pre-Senescent Mice

**DOI:** 10.3390/nu8050246

**Published:** 2016-05-02

**Authors:** Zhi Xia, Jason Cholewa, Yan Zhao, Yue-Qin Yang, Hua-Yu Shang, Lucas Guimarães-Ferreira, Marshall Alan Naimo, Quan-Sheng Su, Nelo Eidy Zanchi

**Affiliations:** 1 Exercise Physiology and Biochemistry Laboratory, College of Physical Education, Jinggangshan University, Ji’an 343009, China; zhixia.ep@hotmail.com (Z.X.); baihu0098@163.com (Y.Z.); 2 Exercise Intervention and Health Promotion Hubei Province Synergy Innovation Center, Wuhan Sports University, Wuhan 430079, China; yangyueqin226@163.com; 3 Department of Kinesiology, Coastal Carolina University, Conway, SC 29528-6054, USA; jcholewa@coastal.edu; 4 Exercise Physiology Laboratory, Department of Exercise Physiology, Beijing Sport University, Beijing 100084, China; santanasan@163.com; 5 Muscle Physiology and Human Performance Research Group, Center of Physical Education and Sports, Federal University of Espirito Santo, Vitória/ES 29075-810, Brazil; lgtrainer@yahoo.com.br; 6 Division of Exercise Physiology, West Virginia University School of Medicine, Morgantown, WV 26506-9227, USA; man03e@my.fsu.edu; 7 Department of Sports Medicine, Chengdu Sport University, Chengdu 610041, China; sqs111@126.com; 8 Department of Physical Education, Federal University of Maranhão (UFMA), São Luís-MA 65020-070, Brazil; 9 Laboratory of Cellular and Molecular Biology of Skeletal Muscle (LABCEMME), São Luís-MA 65020-070, Brazil

**Keywords:** leucine-rich diet, aerobic exercise, aging, protein metabolism

## Abstract

Several studies have indicated a positive influence of leucine supplementation and aerobic training on the aging skeletal muscle signaling pathways that control muscle protein balance and muscle remodeling. However, the effect of a combined intervention requires further clarification. Thirteen month old CD-1^®^ mice were subjected to moderate aerobic exercise (45 min swimming per day with 3% body weight workload) and fed a chow diet with 5% leucine or 3.4% alanine for 8 weeks. Serum and plasma were prepared for glucose, urea nitrogen, insulin and amino acid profile analysis. The white gastrocnemius muscles were used for determination of muscle size and signaling proteins involved in protein synthesis and degradation. The results show that both 8 weeks of leucine supplementation and aerobic training elevated the activity of mTOR (mammalian target of rapamycin) and its downstream target p70S6K and 4E-BP1, inhibited the ubiquitin-proteasome system, and increased fiber cross-sectional area (CSA) in white gastrocnemius muscle. Moreover, leucine supplementation in combination with exercise demonstrated more significant effects, such as greater CSA, protein content and altered phosphorylation (suggestive of increased activity) of protein synthesis signaling proteins, in addition to lower expression of proteins involved in protein degradation compared to leucine or exercise alone. The current study shows moderate aerobic training combined with 5% leucine supplementation has the potential to increase muscle size in fast-twitch skeletal muscle during aging, potentially through increased protein synthesis and decreased protein breakdown.

## 1. Introduction

Aging is a physiological process characterized by progressive decrease in the capacity of organ systems throughout the organism [1]. Of these changes, skeletal muscle is of particular interest as it comprises approximately 40%–50% of human body mass and is highly adaptable [2,3]. Sarcopenia describes the loss of muscle mass and muscle function that occurs during the aging process. When left untreated, sarcopenia substantially contributes to decreased mobility/autonomy and to an increased risk of metabolic diseases. Thus, interventions aimed at maintaining muscle mass and function and attenuating the decline of muscle loss during the aging process are clinically important to sustain an appropriate quality of life and reduce health care costs in the elderly [4].

Exercise training has been considered an important non-pharmacological intervention for the treatment of metabolic disorders and loss of skeletal muscle mass in the elderly [5,6]. Resistance training has been widely recognized as a powerful intervention to maintain or increase skeletal muscle mass [7,8]. Although not considered the most efficient intervention to stimulate a high magnitude of muscle hypertrophy, moderate aerobic exercise has demonstrated anti-catabolic properties in several diseases, like cancer cachexia [9,10,11], cardiac cachexia [11], and diabetes mellitus [12,13], all of which are characterized by a loss of muscle mass. In addition, it has been demonstrated that aerobic exercise increases skeletal muscle protein synthesis (MPS) and strongly activates the mammalian target of rapamycin (mTOR) pathway in healthy subjects [14,15,16]. Therefore, modifications in protein metabolism favoring increased MPS or decreased muscle protein degradation suggests that aerobic training may be an appropriate intervention option to attenuate the development of sarcopenia.

Due to a higher prevalence of nutrient deficiencies in the elderly, nutritional interventions are important and efficacious adjuncts to exercise in the promotion of healthy aging [17]. In particular, several studies have demonstrated higher protein needs in older adults [18,19,20] due to the development of anabolic resistance [20,21]. Of the 20 amino acids, leucine alone has been shown to initiate the translational stage of protein synthesis, and the magnitude of protein synthesis is often directly related to the leucine content of a particular protein source [20]. Moreover, the addition of leucine to a lower protein meal is an easy to consume and effective strategy to increase MPS [14,22]. Although leucine alone is capable of increasing MPS, leucine supplementation combined with resistance training induces a synergistic effect, resulting in increased MPS and myofibrillar muscle hypertrophy in older adults [23]. The predominant muscular adaptation to aerobic training is mitochondrial biogenesis [24], and therefore large changes in myofibrillar protein content have been thought not to occur with leucine consumption in conjunction with aerobic training; however, the effects of age on the interaction between aerobic training and leucine supplementation are not yet known. From a molecular standpoint, both leucine [25] and aerobic exercise [26] have been shown to activate the mTOR pathway, and evidence exists demonstrating that aerobic training [24] and leucine supplementation [27] decreases muscle protein degradation. Considering these anti-catabolic effects reported in different muscle wasting diseases, there is potential for leucine supplementation and aerobic exercise to act synergistically in aging skeletal muscle to attenuate sarcopenia in mice.

Our objective in this study was to investigate the role of leucine supplementation and aerobic training in aging skeletal muscle signaling pathways, controlling muscle protein balance and muscle remodeling.

## 2. Experimental Section

### 2.1. Animals and Diets

Forty 13-month-old CD-1^®^ male mice (47–49 g) were obtained from the Chengdu Dashuo Biological Technology Company (Chengdu, China). All mice were housed in the Laboratory Animal Center of Chengdu Sport University (Chengdu, China). Light and darkness cycles (12–12 h) and temperature (22 ± 1.5 °C) were controlled. Diet and water were available *ad libitum*. Laboratory animal bedding materials were renewed 3 times a week; the drinking bottles were disinfected daily. Animal welfare and experimental procedures were carried out in accordance with the national and institutional guidelines, and the study was reviewed and approved by the Institutional Animal Ethics Committee of Chengdu Sport University, number: 2015/03.

The non-purified diet based on maize, wheat flour, wheat bran, soybean flour, soya bean meal, rice bran, and fish meal was purchased from the Institute of Experimental Animals, Sichuan Provincial Academy of Medical Sciences (Chengdu, China). 5% (*w/w*) l-leucine was added to the intervention diet by replacing equal amounts of corn flour, while the isonitrogenous control diet contained an added 3.4% (*w/w*) l-alanine (Table 1). The l-alanine-rich meal was used as the control diet in light of previous reports that this non-essential amino acid does not affect muscle protein metabolism [28,29,30,31].

The supplemental dose of 5% l-leucine was used because it has been shown in previous research to positively regulate the protein synthesis of skeletal muscles [30,31]. Nutrient levels of the non-purified meal were digestible energy (14.32 MJ/kg), protein (20.6%, *w/w*), Ca (1.35%, *w/w*), total P (1.20%, *w/w*) and available P (0.97%, *w/w*). The analyzed contents (%, *w/w*) of amino acids in the leucine-supplemented and alanine-supplemented meal are summarized in Table 2.

### 2.2. Experimental Protocols

Forty mice were randomly assigned to four groups with ten mice per group: animals were fed an alanine-supplemented non-purified diet without exercise intervention (AlaC) or with moderate physical exercise (AlaE). The other two groups were fed the leucine-supplemented diet combined with (LeuE) or without (LeuC) exercise, respectively. Body weight and food intake were recorded every day. The mice were housed in individual cages throughout experimentation. When we renewed the bedding materials the 6th week, some mice were severely bitten on the face or limbs. Therefore, to avoid interference of accelerated protein synthesis during the recovery period of injury, the injured animals were all excluded from the experiment. Thus, only the 33 unharmed mice were included in the final statistical analysis.

Mice in the AlaE and LeuE groups received moderate intensity swimming exercise in two glass tanks (100 × 70 × 60 cm) filled with 30 ± 2 °C water to 40–45 cm depth at once so that the mice could not have a rest by getting their tail on the bottom of the tank [32]. The exercise protocol consisted of swimming exercise (45 min/day, 6 day/week) for 8 weeks, with a 3% bodyweight workload that corresponds to an aerobic intensity of approximately 10%–20% below the anaerobic threshold in 10–12 month old mice. We employed this intensity as it is more reasonable to expect elderly humans to work at a lower intensity and not near the anaerobic threshold. The loads were readjusted each day according to the bodyweight of the mice. A monitor was arranged to confirm that all mice were actually swimming and not engaged in behaviors that minimized the effort exerted, such as floating. Once the negative activities were observed, the monitor forced the animals to keep swimming with a glass bar. We randomly measured the blood lactate concentration from the tail vein of mice in the AlaE and LeuE groups during and immediately after swimming exercise by using a portable blood lactate analyzer (Lactate pro, Arkray, Kyoto, Japan), and all concentrations were between 2.9 and 3.7 mmol/L. This suggested that the exercise protocol used was typical of aerobic exercise bouts that may be employed in elderly humans where blood lactate values have been reported as 2.44 ± 1.04 mmol/L during aquatic aerobics [33].

Twenty-four hours following the final bout of exercise (7 h food deprivation), animals were euthanized with intraperitoneal injection of pentobarbital sodium (80 mg/kg), after which blood was collected from the aortaventralis. The blood samples were then centrifuged at 12,000 rpm for 10 min at 4 °C to obtain plasma, or placed at room temperature for 0.5 h and centrifuged at 5000 rpm for 10 min to obtain serum. The superficial portion (white) of gastrocnemius muscles was separated and washed with PBS, and then the muscle samples were dried using filter paper. After that, samples were weighed and frozen by liquid nitrogen for western blotting analysis. Specimens from the contralateral white gastrocnemius were fixed for histological examination.

### 2.3. Blood Glucose, Serum Insulin, Serum Urea Nitrogen, and Plasma Free Amino Acids Measurement

The blood glucose level was measured from the tail vein of mice using a glucometer (Johnson OneTouch UltraEasy, Shenzhen, China). The concentration of serum insulin was determined using a mouse insulin ELISA kit (Mercodia Diagnostics, Uppsala, Sweden). Serum urea nitrogen analysis was performed using a commercial kit (Jiancheng Bioengineering Institute, Nanjing, China). Free amino acids in plasma were analyzed using the Hitachi L-8800 amino acid analyzer (Hitachi, Tokyo, Japan) after acid hydrolysis with 6N HCl (refluxed at 110 °C for 24 h) [34].

### 2.4. Western Blot Analysis

Fifty mg of frozen white gastrocnemius was powdered and homogenized for 30 s in 0.5 mL lysis buffer (containing protease inhibitors with or without phosphatase inhibitors, accordingly, to detect the expression of phosphorylated or non-phosphorylated protein). The homogenate was incubated on ice for 20 min and then centrifuged at 13,000× *g* at 4 °C for 20 min. Supernatants were carefully transferred to an Eppendorf tube and stored at −80 °C until used for western blots. The protein concentration in muscle homogenate was determined with a BCA protein assay kit (Thermo Scientific, Rockford, IL, USA). A total of 20 μg of protein was loaded per lane, separated by SDS-PAGE (8% or 12%) and transferred to nitrocellulose filter membranes (NC, 0.45 μm). The membranes were blocked with 3% BSA-TBST for 30 min and incubated overnight at 4 °C with mammalian vacuolar protein sorting mutant 34 (mVPS34, 1:1000 dilution), mTOR (1:1000), phospho-mTOR (Ser2448) (1:1000), 70kDa ribosomal protein S6 kinase (p70S6K, 1:2000), phospho-p70S6K (Thr389) (1:500), eukaryotic translation initiation factor 4E-binding protein 1 (4E-BP1, 1:2000), phospho-4E-BP1 (Thr37/46) (1:1000), fast myosin skeletal heavy chain (MHC II, 1:3000), Ubiquitin (1:1000), muscle RING-finger protein-1 (MuRF-1, 1:1000), and muscle atrophy F-box (MAFbx/Atrogin-1, 1:1000) antibodies (Cell Signaling Technology, Beverly, MA, USA or Abcam, Cambridge, UK) at different dilutions. The next day, the membranes were incubated at room temperature for 30 min and washed 5 times with tris-buffered saline with tween 20 (TBST), then incubated with secondary antibody (Jackson Immuno Research, Laboratories, Inc., West Grove, PA, USA) anti-rabbit (1:20,000) or anti-mice (1:10,000) for 40 min. Blots were serially washed six times and detected with ECL reagent kit (Merck Millipore, Billerica, MA, USA). The optical density blotting was analyzed using Totallab (Nonlinear Dynamics, Newcastle, UK) [35]. The comparisons of both protein expression and phosphorylation rate between groups were performed with the raw data. The differences were displayed with the relative value, which was normalized to a control (AlaC).

### 2.5. Histological Examination

The specimens of white gastrocnemius were fixed with 4% (*v/v*) paraformaldehyde in 0.01 M PBS (pH 7.4) for 3 h and embedded in paraffin. Five μm sections were cut and then stained using a standard haematoxylin and eosin staining method. The images were captured with a 40× objective on the Olympus DP71 microscope digital camera (Olympus; Tokyo, Japan) and analyzed using Image-Pro Plus version 6.0 software (Media-Cybernetics, Silver Spring, MD, USA) to measure the cross-sectional area (CSA) and diameter of muscle fibers [36].

### 2.6. Statistical Analysis

The normality of the data was checked and subsequently confirmed with the Shapiro–Wilk test. The Levene’s homogeneity of variance test was used to assess the equality of variances; when Levene’s test was significant, the Greenhouse–Geisser correction was used. Data that were not normally distributed were then transformed and retested. Following transformations, data that failed to meet the assumptions of normality and homogeneity of variances were analyzed with non-parametric tests. Data are expressed as means plus or minus the standard deviation. When measurements could be repeated over time (body weight, average daily food intake), repeated measures analysis of variance was performed. The data of other variables were all analyzed by univariate and two-factor analysis of variance. When a significant interaction effect was detected, the simple effect was assessed with the results of one-factor analysis of variance. The significance of the differences between groups was assessed by Dunnett’s correction for multiple comparisons. All tests were performed using statistical software SPSS version 20.0 (SPSS, Inc., Chicago, IL, USA), and all figures were created with Sigmaplot 11.0 (Systat, San Jose, CA, USA). Differences were considered as significant for *p <* 0.05.

## 3. Results

### 3.1. Body Weight

**Figure 1:**
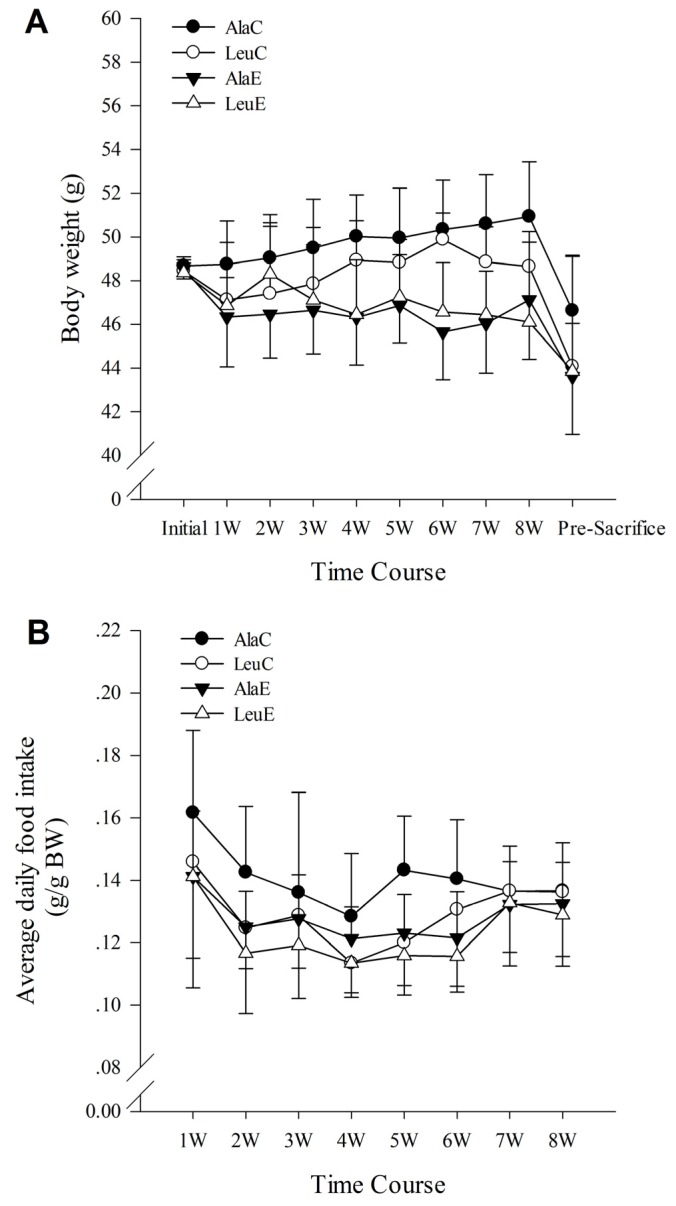
(**A**) Effects of leucine supplementation and exercise on body weight (BW); and (**B**) food intake. AlaC: alanine supplementation group; LeuC: leucine supplementation group; AlaE: alanine supplementation + exercise training group; LeuE: leucine supplementation + exercise training group.

The body weight of mice in the two sedentary groups (AlaC, LeuC) were both elevated after 8 weeks when compared with their initial weight; however, only the AlaC group reached statistical significance (*p <* 0.05). In comparison, the body weight of mice in the exercise groups did not change significantly (AlaE, *p =* 0.132; LeuE, *p =* 0.136). After the 7 h food deprivation, the body weight of all mice except LeuE was significantly reduced. Repeated measures ANOVA for data in the 8 week experimental period revealed that body weight did not vary with time and the effects of time (week) did not vary among each group. The multiple comparisons between eight time-points within each group also showed that the body weight of all mice were stable. A main effect of the intervention was found to significantly influence body weight (*p <* 0.01) (Figure 1A).

### 3.2. Food Intake

A significant main effect was found for time (*p <* 0.05). There were no significant interactions, suggesting that the average daily food intake of the mice in each group was consistent between groups throughout the 8 week intervention period (Figure 1B).

### 3.3. Concentrations of Serum Insulin, Urea Nitrogen, and Blood Glucose

**Figure 2:**
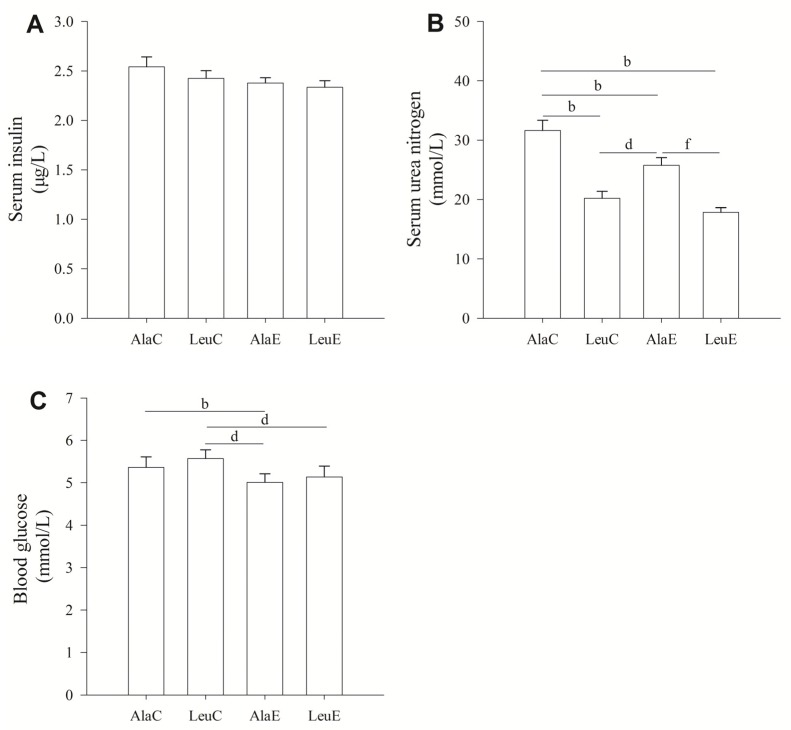
(**A**) Effects of leucine supplementation and exercise on serum insulin; (**B**) serum urea nitrogen; and (**C**) blood glucose in pre-senescent mice. AlaC: alanine supplementation group; LeuC: leucine supplementation group; AlaE: alanine supplementation + exercise training group; LeuE: leucine supplementation + exercise training group. Values are provided as mean ± standard deviation for each group (*n* = 7–10). a: *p <* 0.05 *versus* the AlaC group; b: *p <* 0.01 *versus* the AlaC group; c: *p <* 0.05 *versus* the LeuC group; d: *p <* 0.01 *versus* the LeuC group; e: *p <* 0.05 *versus* the AlaE group; f: *p <* 0.01 *versus* the AlaE group.

There were no significant effects of the different interventions on serum insulin. The concentration of serum urea nitrogen was affected by both leucine supplementation and exercise training (*p <* 0.01); however, there were no significant interactions. Compared to AlaC, serum urea nitrogen significantly decreased in the LeuC, AlaE, and LeuE groups. *Post hoc* analysis revealed that LeuC and LeuE were both significantly less than AlaE. Blood glucose concentration was significantly greater (*p <* 0.01) in AlaC compared to AlaE, and LeuC was significantly greater (*p <* 0.01) than LeuE. Exercise significantly reduced blood glucose concentrations; however, there were no differences between AlaC and LeuC (Figure 2).

### 3.4. Plasma Free Amino Acids Analysis

Regarding the main effects, the factor of amino acid supplementation only significantly affected leucine, alanine, proline, glutamic acid, glycine, and tyrosine, while exercise had more extensive effects on the concentrations of leucine, isoleucine, arginine, histidine, threonine, alanine, serine, glutamic acid, glycine, and tyrosine. A significant interaction between amino acids and exercise were observed in glutamic acid, glycine, and tyrosine.

Plasma concentrations of all essential amino acids except leucine were not significantly affected by the different interventions. The leucine concentration in LeuC, AlaE, and LeuE was higher than AlaC, with the greatest values being in LeuE (132%), followed by LeuC (124%) and AlaE (116%). In regard to non-essential amino acids, concentrations of alanine, glutamic acid, glycine, and tyrosine were significantly different. In the LeuE group, alanine and tyrosine concentrations were higher than mice in AlaC (Ala 123%, Tyr 128%) and LeuC (Ala 147%, Tyr 134%), but lower than that in AlaE. Plasma glutamic acid and glycine were lower in LeuE than AlaC and AlaE (Table 3).

### 3.5. Histological Changes of Muscle Fibers

**Figure 3:**
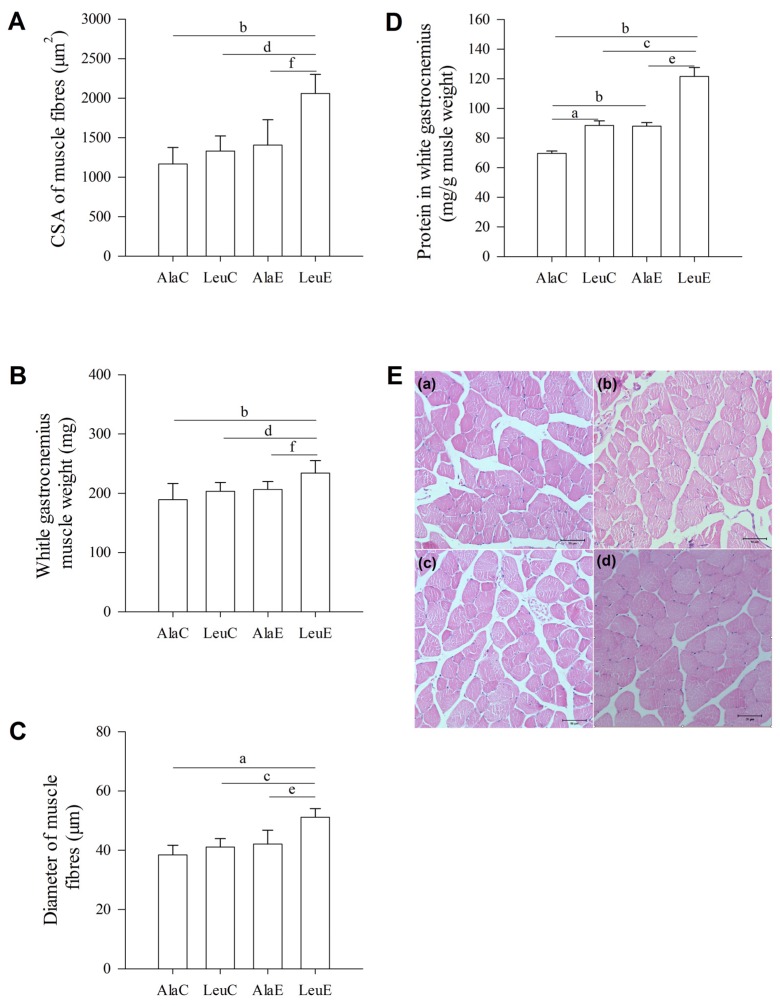
Effects of leucine supplementation and exercise on (**A**) cross-sectional area (CSA), (**B**) diameter, (**C**) weight, and (**D**) protein ratio; (**E**) Hematoxylin-eosin stained sections of white gastrocnemius muscle in mice: (**a**) Cross-section of alanine supplementation; (**b**) leucine supplementation; (**c**) alanine supplementation + exercise training; and (**d**) leucine supplementation + exercise training mice. Magnification 400×, scale bars = 50 μm. Values are provided as mean ± standard deviation for each group (*n* = 7–10). AlaC: alanine supplementation group; LeuC: leucine supplementation group; AlaE: alanine supplementation + exercise training group; LeuE: leucine supplementation + exercise training group. a: *p <* 0.05 *versus* the AlaC group; b: *p <* 0.01 *versus* the AlaC group; c: *p <* 0.05 *versus* the LeuC group; d: *p <* 0.01 *versus* the LeuC group; e: *p <* 0.05 *versus* the AlaE group; f: *p <* 0.01 *versus* the AlaE group.

Compared with the control group (AlaC), histological examination of haematoxylin and eosin stained white gastrocnemius sections showed significant increases (*p <* 0.01) in CSA (Figure 3A) and diameter (Figure 3C) of the muscle fibers in the LeuE group, whereas the CSA and diameter of these fibers were not changed in LeuC and AlaE (Figure 3E). A main effect of group was found for gastrocnemius weight (*p <* 0.05). Gastrocnemius muscle weight was significantly greater (*p <* 0.01) in LeuE than LeuC, AlaE, and AlaC (Figure 3B).

### 3.6. Total Protein in White Gastrocnemius Muscle

Muscle protein content is reported as mg muscle∙g body weight^−1^ [37]. When compared with AlaC, the content of white gastrocnemius muscle protein in LeuC, AlaE, and LeuE were all significantly greater (*p <* 0.01). The differences between LeuC and LeuE, and AlaE and LeuE were also significant (*p <* 0.01, Figure 3D). The protein content in LeuE was greater than LeuC, AlaE, and AlaC (*p <* 0.01).

### 3.7. Protein Expression with Relation to Hypertrophy and Atrophy

**Figure 4:**
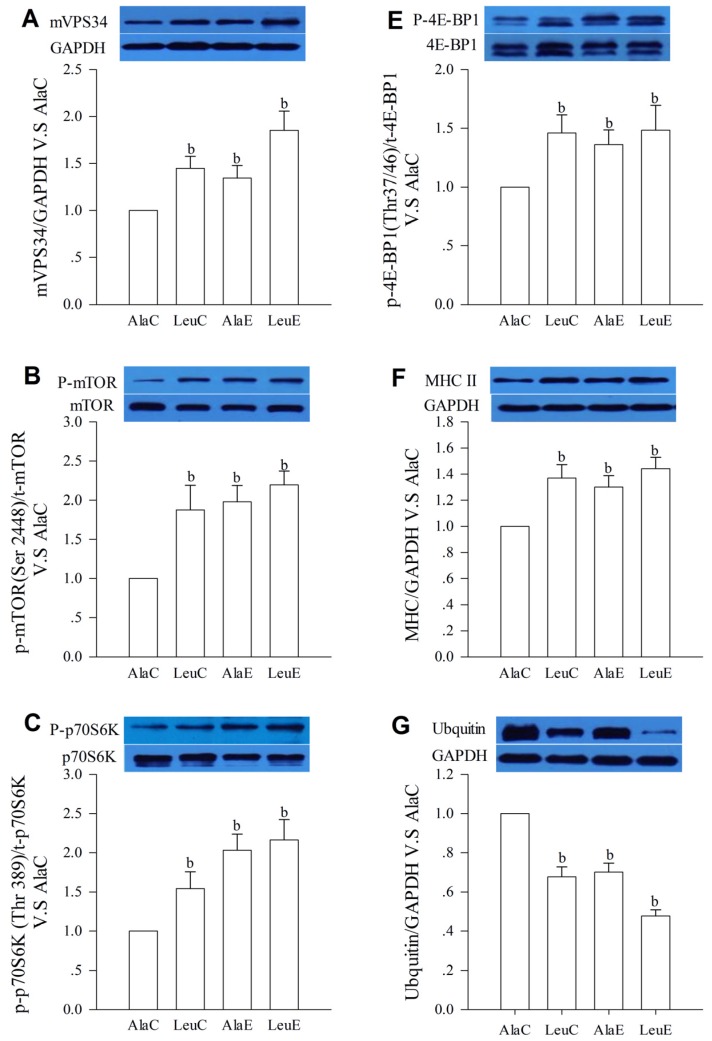

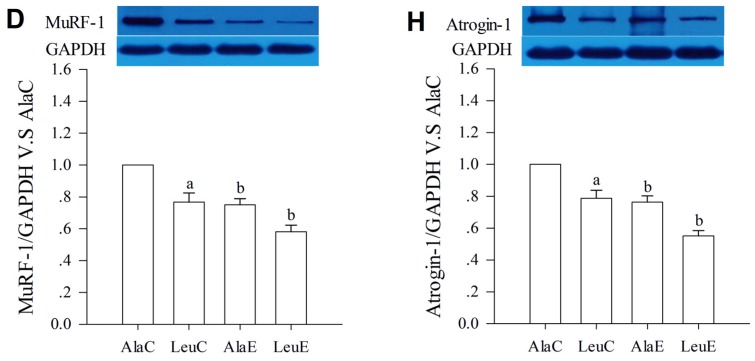
Effects of leucine supplementation and exercise on protein expression with relation to hypertrophy and atrophy. AlaC alanine supplementation group, LeuC leucine supplementation group, AlaE alanine supplementation + exercise training group, LeuE leucine supplementation + exercise training group. (**A**) mVPS34; (**B**) mammalian target of rapamycin (mTOR); (**C**) p70S6K; (**D**) MuRF-1; (**E**) 4E-BP1; (**F**) MHC; (**G**) Ubiquitin; and (**H**) Atrogin-1. Values are provided as mean ± standard deviation for each group (*n* = 7–10). a: *p <* 0.05 *versus* the AlaC group; b: *p <* 0.01 *versus* the AlaC group.

AlaE, LeuC, and LeuE all increased (*p <* 0.01) the protein expression of mVPS34, MHC II and the phosphorylation state ratio of mTOR (Ser2448), p70S6K (Thr389), and 4E-BP1 (Thr37/46) compared to AlaC. The main effects of both leucine supplementation and exercise were statistically significant for all proteins. A significant interaction between these two factors was not found. AlaE, LeuC, and LeuE all decreased (*p <* 0.05 or *p <* 0.01) the protein expression of Ubiquitin, MuRF-1, and Atrogin-1, which are involved in the ubiquitin-proteasome pathway. Both the leucine and exercise factor showed statistically significant main effects (*p <* 0.05); however, no significant interaction was found (Figure 4).

## 4. Discussion

Aging is a multifactorial process characterized by decreased protein synthesis and increased protein breakdown, which, in conjunction with reduced food/protein intake and activity, contributes to skeletal muscle atrophy and sarcopenia [1,38].

The enhanced muscle CSA, protein content, and altered phosphorylation (suggestive of increased activity) of protein synthesis signaling proteins, and the decreased expression of proteins involved in protein degradation observed in LeuC, AlaE, and LeuE in the present study may have been due to increased plasma amino acid concentrations, as only fasting plasma free leucine differed between groups. In this regard, both leucine supplementation and exercise training elevated leucine concentrations in the fasting state. This elevation observed in the leucine group was not surprising, given that modifications in muscle branched-chain amino acid (BCAA) metabolism is frequently reported with leucine supplementation [27]. However, exercise training also produced a similar effect [39,40]. This may have been due to reduced leucine uptake by skeletal muscle, as sufficient energy intake and available glucose leads to reduced leucine oxidation in skeletal muscle [10]. Although increased plasma leucine (and BCAA) levels has been associated with metabolic diseases such as obesity and type 2 diabetes [27,41], our data does not point to a glucose homeostasis disturbance, as presented in the Figure 2A, C. Although leucine supplementation and exercise training was associated with increased plasma concentration of several amino acids, this may have been due to the fasting process or as a result of leucine supplementation. We can not conclude that the protein breakdown was accelerated due to increased plasma alanine during the fasting state alone. Moreover, alanine concentrations were greater in AlaE compared to LeuE. This suggests that if the increased plasma alanine during the fasting period was to provide substrate for gluconeogenesis, leucine supplementation may attenuate this breakdown. The histological changes, protein content of muscles, and serum urea nitrogen (see below) further suggests that protein metabolism was positively regulated when moderate aerobic exercise was accompanied by leucine supplementation.

Both leucine [14,22] and exercise [7,8] have been shown to affect the mTOR pathway in aging mice. mTOR integrates the input from multiple upstream signaling pathways, including mechanical and nutritional stimulation, to regulate several eukaryotic cellular functions, such as protein synthesis [42]. The up-regulation of protein synthesis after leucine supplementation and exercise training is the result of the activation of the mammalian target of rapamycin complex 1 (mTORC1) and its down-stream targets p70S6K and 4E-BP1 [14]. Although chronic low-intensity endurance exercise generally does not provide enough mechanical overload to induce significant increases in muscle mass, higher-intensity aerobic exercise training was found to increase muscle protein synthesis and mTOR signaling in older adults [43]. Konopka and Harber suggest that the effectiveness of aerobic exercise training in inducing skeletal muscle hypertrophy most likely depends on obtaining a sufficient exercise intensity (70%–80% heart rate reserve, HRR). However, this intensity may not be appropriate for some older adults, such as elderly individuals with osteoarthritis, pre-existing injuries, or the frail. Considering the potential impact of "anabolic resistance" to protein feeding, we assume that leucine combined with lower intensity aerobic exercise training may confer benefits that otherwise would require higher-intensity aerobic exercise. Our study demonstrates that pre-senescent mice benefit from aerobic exercise, as evidenced by muscle remodeling evaluated through histological and biochemical methods. In the present study, we observed a significant increase in CSA and the ratio of protein to body weight of LeuE *versus* AlaC mice. Interestingly, 5% leucine supplementation without exercise also showed similar effects, as the protein content of white gastrocnemius was elevated by 34% in LeuC *versus* AlaC. This is consistent with the findings of previous research that have examined the effects of a leucine-rich diet on protein synthesis [30,31].

The key upstream proteins of mTOR in the amino acid sensing pathway require further clarification. Amino acids do not appear to signal through Akt to activate mTORC1 [44]. Instead, these nutrients signal through other kinases to mTORC1. The Class III PI3K, mammalian vacuolar protein sorting mutant 34 (mVPS34) has been found to play a critical role in mTORC1 activation [45,46]. MacKenzie *et al.* [45,46] found that resistance exercise or high-resistance contractions activated mVPS34, but the effects of aerobic exercise on mVPS34 were not reported. In the present study, the protein expression of mVPS34 was up-regulated by leucine supplementation and aerobic exercise training, but there were no significant interactions between these two interventions. Moreover, the phosphorylation of mTOR, p70S6K, and 4E-BP1 were all increased in LeuC, AlaE, and LeuE mice compared to controls. These expression changes suggest that protein synthesis in white gastrocnemius is promoted by leucine supplementation and exercise. Additionally, leucine supplementation without exercise increased protein expression associated with protein synthesis similarly to those achieved with aerobic exercise.

Skeletal muscle fiber type and contractile function are determined by myosin heavy chain (MHC) isoforms [47,48]. Contractile phenotype in skeletal muscle fibers is primarily determined by the relative expression of the MHC isoforms I and II [49]. There is a decrease in the fractional content of MHC II protein in skeletal muscle, and a corresponding increase in the proportion of MHC I with age, and this change is consistent with the reduction of CSA occupied by histochemically-typed fast-twitch fibers in skeletal muscle [50]. We found the protein expression of MHC II, wet weight, diameter, and CSA of white gastrocnemius was greater for LeuE compared to LeuC, AlaE, and AlaC; however, there were no differences between LeuC, AlaE, or AlaC. On the other hand, protein concentrations of white gastrocnemius in LeuC, AlaE, and LeuE mice were significantly increased compared with those of the control group. Moreover, the CSA and total protein per muscle fiber was the highest in LeuE. These changes may further suggest that leucine supplementation in conjunction with aerobic exercise training promotes protein accretion in white gastrocnemius, without affecting variations in skeletal muscle.

The ubiquitin-proteasome system (UPS) is the primary pathway regulating proteolysis in skeletal muscle and plays a critical role in the control of protein degradation in muscle fibers [51,52]. UPS-dependent protein degradation is known to be highly regulated by exercise and nutrient availability [53,54]. Two muscle-specific E3 ubiquitin ligases, Atrogin-1 and MuRF1, are considered to be critical components involved in these regulatory effects [55,56]. Therefore, UPS plays a major role in controlling skeletal muscle mass in aging [52]. The skeletal muscle atrophy through UPS arises in the pre-senescent state and shows progressive increases in lean body mass loss with age. In the present study, mice receiving leucine supplementation and exercise training showed a decrease in protein expression of ubiquitin, Atrogin-1, and MuRF-1, and the combination of leucine and exercise led to more significant decreases compared with exercise or leucine supplementation alone. We previously demonstrated diminished gene expression of Atrogin-1 and MuRF-1 after a chronic program of resistance training in rats [57]. This study demonstrates for the first time that aerobic training or leucine supplementation is also capable of inhibiting both atrogenes, with a more significant inhibition observed when leucine supplementation is combined with aerobic training. The inhibition of UPS components with leucine and exercise suggests that protein degradation in pre-senescent mice was decreased, and therefore may play a role in maintaining skeletal muscle mass. Additionally, serum urea nitrogen was also lower in the leucine and exercise group suggesting improved nitrogen balance. Therefore, the greater muscle mass we observed in LeuE compared to AlaC is likely most attributable to the potential synergistic effects of leucine supplementation and exercise on inhibiting muscle protein breakdown.

## 5. Conclusions

In conclusion, long-term leucine supplementation, swimming exercise training, and combined interventions are potential treatments to enhance white gastrocnemius mass in pre-senescent mice, with the combination of leucine and exercise showing the greatest effects. These results may have been achieved by stimulating protein synthesis, but are predominantly attributable to inhibiting muscle protein degradation. Interestingly, in our sample of mice, leucine supplementation alone showed similar effects to aerobic training; however, more human data is needed to translate these effects. From a molecular mechanistic standpoint, mVPS34/mTOR and UPS may be involved in these regulatory effects induced by leucine and/or exercise, and the leucine availability may also play a central role in this process. Further research is needed to investigate the use of inhibitors or blockers of these critical signaling proteins (*i.e.*, mVPS34, mTOR, Atorgin-1, and MuRF-1), as they were not utilized in this work. Future research should also investigate the dose–effect relationship of leucine supplementation and exercise training (including intensity and duration) in aging populations. Finally, we acknowledge that this research was limited by the lack of muscle function testing, because increased muscle mass has not always positively correlated with muscle function. Thus, the effects of different interventions on measures of muscle function such as maximal force output and peak power will be potentially fruitful areas of future research.

## Figures and Tables

**Table 1:** Diet formulation (%).

Feed Composition	Basal Diet	Leucine Diet	Alanine Diet
Corn	22.8	17.8	19.4
Wheat	34.0	34.0	34.0
Wheat bran	10.0	10.0	10.0
Soybean	13.0	13.0	13.0
Soya bean meal	5.0	5.0	5.0
Rice bran	4.0	4.0	4.0
Fish meal	7.0	7.0	7.0
Calcium hydrogen phosphate I	2.4	2.4	2.4
Calcium carbonate	0.6	0.6	0.6
Additives and Microelements	1.2	1.2	1.2
Leucine	~	5.0	~
Alanine	~	~	3.4

**Table 2:** Analyzed contents of amino acids (g/100 g) in the alanine- and leucine-supplemented.

Amino Acid Composition	Basal Diet	Leucine Diet	Alanine Diet
Aspartic acid	1.81	1.72	1.72
Threonine	0.71	0.70	0.71
Serine	0.97	0.94	0.94
Glutamic acid	4.06	4.05	4.01
Glycine	0.97	0.92	0.93
Alanine	1.11	1.04	4.83
Cystine	0.11	0.20	0.17
Valine	0.78	0.79	0.82
Methionine	0.45	0.54	0.50
Isoleucine	0.64	0.62	0.62
Leucine	1.45	6.51	1.42
Tyrosine	0.39	0.47	0.46
Phenylalanine	0.79	0.86	0.86
Lysine	1.17	1.10	1.15
Histidine	0.69	0.62	0.71
Argnine	1.13	1.06	1.04
Proline	1.39	1.30	1.38

**Table 3:** Mean plasma amino acids concentrations after 8 weeks of leucine supplementation with/without exercise training (μmol/L).

	AlaC (*n =* 8)	LeuC (*n =* 7)	AlaE (*n =* 10)	LeuE (*n =* 8)
Essential amino acids				
Leucine	127.3 ± 7.0	158.8 ± 5.5 ^b^	148.0 ± 9.2 ^b,c^	168.5 ± 11.1 ^b,f^
Isoleucine	74.6 ± 2.7	73.2 ± 3.2	78.7 ± 2.7	77.7 ± 6.0
Valine	281.0 ± 9.2	268.2 ± 30.7	279.7 ± 2.6	269.2 ± 16.4
Arginine	71.5 ± 9.2	69.9 ± 5.6	78.1 ± 4.3	76.5 ± 2.5
Histidine	125.1 ± 6.6	126.8 ± 8.4	132.1 ± 3.4	132.4 ± 4.5
Lysine	269.2 ± 6.4	270.6 ± 12.0	277.1 ± 14.3	282.5 ± 12.5
Methionine	50.7 ± 8.5	54.1 ± 1.0	56.1 ± 9.3	59.1 ± 9.2
Phenylalanine	252.2 ± 14.8	261.5 ± 16.1	266.4 ± 12.8	268.0 ± 7.0
Threonine	140.9 ± 7.1	141.2 ± 12.0	148.9 ± 11.4	152.6 ± 4.9
Non-essential amino acids				
Alanine	306.3 ± 33.1	256.0 ± 23.3 ^b^	415.7 ± 31.9 ^b,d^	378.2 ± 21.1 ^b,d,e^
Aspartic acid	147.7 ± 13.5	151.4 ± 11.8	148.4 ± 6.6	158.3 ± 14.3
Cystine	47.5 ± 5.3	47.8 ± 9.3	51.2 ± 2.4	53.0 ± 5.3
Glutamic acid	465.2 ± 28.5	390.8 ± 20.2 ^b^	549.3 ± 38.0 ^b,d^	406.1 ± 26.4 ^b,f^
Glycine	380.6 ± 25.4	349.3 ± 13.8 ^a^	442.0 ± 24.5 ^b,d^	365.7 ± 15.7 ^f^
Proline	103.3 ± 3.6	107.5 ± 6.1	100.2 ± 4.9	107.1 ± 8.1
Serine	202.2 ± 13.2	198.9 ± 7.5	210.6 ± 9.1	210.4 ± 11.2
Tyrosine	94.1 ± 5.7	89.4 ± 6.8	148.5 ± 8.8 ^b,d^	120.5 ± 10.8 ^b,d,f^

Values are provided as mean ± standard deviation for each group (*n* = 7–10). AlaC: alanine supplementation group; LeuC: leucine supplementation group; AlaE: alanine supplementation + exercise training group; LeuE: leucine supplementation + exercise training group. Glutamic acid shown here includes both glutamine and glutamic acid. a: *p <* 0.05 *versus* the AlaC group; b: *p <* 0.01 *versus* the AlaC group; c: *p <* 0.05 *versus* the LeuC group; d: *p <* 0.01 *versus* the LeuC group; e: *p <* 0.05 *versus* the AlaE group; f: *p <* 0.01 *versus* the AlaE group.
